# Satellite radar observation of large surface collapses induced by the 2017 North Korea nuclear test

**DOI:** 10.1038/s41598-020-74957-2

**Published:** 2020-10-20

**Authors:** Won-Kyung Baek, Hyung-Sup Jung, Tae Sung Kim

**Affiliations:** 1 Department of Geoinformatics, University of Seoul, Seoul, Korea; 2 Earthquake Research Centre, Korea Institute of Geoscience and Mineral Resources, Seoul, Korea

**Keywords:** Geology, Environmental social sciences, Solid Earth sciences

## Abstract

The artificial earthquake of mb 6.1 related to the North Korea’s sixth nuclear test occured at Mt. Mantap, North Korea on September 3, 2017. It was reported that a large and complex surface deformation was caused by the event. The surface deformation was composed of expansion of explosions, collapse, compaction and landslides. Since the precise vertical deformation measurement is very important to estimate the stability of the nuclear test facility, we retrieved a precise 3D surface deformation field and then decomposed the vertical deformation pattern from the 3D deformation. The measured maximum deformation was about − 491, − 343 and 166 cm with the measurement uncertainty of about 3.3, 4.1 and 2.7 cm in the east, north and up directions, respectively. The maximum horizontal deformation was approximately 515 cm. The horizontal deformation clearly showed a radial pattern because it was mainly caused by the explosions and landslides, while the vertical deformation displayed a rugged pattern because it was affected by the explosions, compaction and collapse. The collapse may seem to occur along the underground tunnels and at the test site’s epicenter as well. Moreover, the severe collapse was observed westside from the epicenter of the sixth nuclear test, and it has a depth of about 68.6 cm on the area of 0.3765 km^2^. On the basis of our results including the shapes, locations and volume changes of the large collapse, evidently a new vital piece of information was obtained so that it could be used to interprete the sixth nuclear test more accurately.

## Introduction

North Korea has conducted six nuclear tests at Mt. Mantap, Pungyeri, North Korea since 2006. The seismic magnitudes estimated from the North Korean’s nuclear tests has been increased since it’s first test. The magnitude of the sixth test was mb 6.1, which is the biggest one among the six tests. Due to North Korea’s sixth underground nuclear tests, large and complex deformations occurred, which also caused landslides around the test site^1–3^. After 8 min 32 s from the sixth test, the second earthquake of smaller magnitude occured^1,2^. Many research groups including USGS have considered the source of second earthquake as a large collapse, which also raised up questions on the stability of the infrastructures of the test site under the Mt. Mantap. The infrastructures in seismically active area usually experience irrecoverable damages and ground subsidence^4–6^. Measuring surface subsidence could be used to estimate the ground stablility^6,7^. However, there have been no valid measurements about the collapse or ground subsidence in the North Korea Nuclear test site because of a paucity of information about the North Korea nuclear test^8,9^.

Synthetic aperture radar (SAR) is a unique method that can generate deformation map with milimeter to centimeter precision^10–13^. A lot of geological events have been measured by using SAR Interferometry (InSAR) or offset tracking (OT) methods such as volcano^14–16^, earthquakes^14,17,18^, landslides^19,20^, land subsidence^21,22^ and glaciers^23–26^. Among them, land subsidence is especially successful and traditional application of SAR deformation measurement^27–31^. For example, risk assesments of underground voids(mines, tunnels) were performed via multiple-temporal InSAR. Falorni et al. introuduced surface deformation rate variation according to mining and rail tunnel excavation progression^30^. Rabus et al. monitored land subsidence on the subway and water pump tunnels^31^. Since InSAR was firstly applied to Nevada nuclear test site to measure the surface deformation, several studies have been reported about observing the nuclear test sites using InSAR^8,32–34^. Wang et al. (2018) observed the deformation from the 2017 North Korea nuclear test using SAR measurements and suggested a reasonable deformation scenario^1^. However, there have been no deformation analysis about large surface collapses induced by the 2017 North Korea Nuclear test. In order to measure large collapses in North Korea’s test site, three main issues need to be considered.

First, InSAR measurements near the test site was too decorrelated to map the deformation^1,2,8,35–37^. The biggest explosion inevitably caused complex and steep surface deformation which exceeds the phase difference of 2 $\uppi$ between adjacent pixels^1,8,36–38^. Thus, InSAR could not be utilized to describe the deformation nearby the test site^34^. The OT method is an alternative aprpoach to measure the large and complex surface deformation induced by the 2017 North Korea Nuclear test because the method is allowed to measure the complex and steep surface deformation by using intensity cross-correlation approach^26,36–38^.

Second, the subsidence from the second event was known as occupying only localized area of the deformation fields^1^. Therefore, the spatial resolution and precision of the measurements are very important to recognize the spatial distribution of the ground subsidence. The precision and resolution of the OT measurements from a single search kernel largely depends on the search kernel size^23,25,39^. When the window size is small, the detailed correlation peak could be estimated well, therefore the resolution will be higher. However, in case the search kernel size is too small, the measurement precision will be much lower. On the other hand, if the kernel size is larger, the number of effective measurements must increase, but the resolution will be much lower. The trade-off relation needs to be overcome. In case of Wang et al. (2018), they generated OT measurements with large kernel to enhance measurement precision, since they focused on estimating explosive source using SAR data. Thus, their measurements were too smoothed to recognize the surface collapses^1^.

Third, three-dimensional (3D) decomposition were essential to measure the localized land subsidence^1,40,41^. The geometry of the OT measurements from the one path SAR represents two-dimensional (2D) deformations in the range and azimuth directions, which is respectively line-of-sight (LOS) and along-track (AT) deformations. The LOS measurement includes both the horizontal and vertical components of surface deformation^25,40–42^. Thus, the vertical deformation should be decomposed from the LOS deformation, because the horizontal and vertical deformations were complexly mixed in the LOS deformation^1^.

In this study, we show the evidence of large collapse induced by the 2017 North Korea Nuclear test. For that, a multi-kernel OT (MKOT) approach was employed to overcome the limitation of the trade-off between measurement precision and spatial resolution^36–38^. The two ALOS PALSAR-2 stripmap interferometric pairs were acquired from the ascending (20170829_20170912) and descending (20170831_20170928) orbits, and the pairs were utilized to measure the ascending and descending LOS and AT deformations. Then 3D deformation field was retrieved from the ascending and descending LOS and AT deformations. Finally, the spatial patterns of the horizontal and vertical deformations were separately analyzed.

## Results

### Study area and data

**Figure 1:**
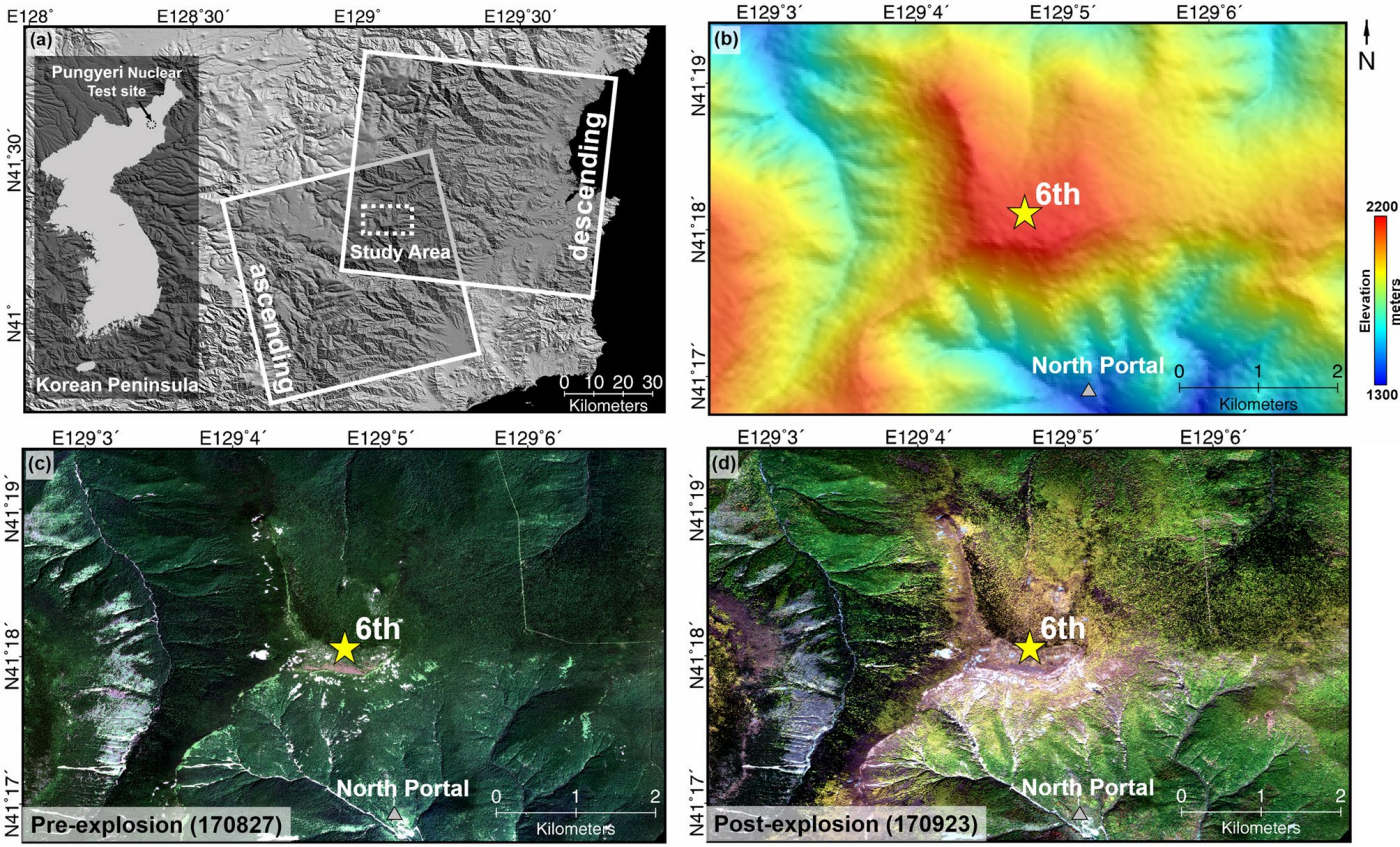
The nuclear test site located in the basement of the Mt. Mantap at Punggye-ri, North Korea; **(a)** shadow relief map of the Pungyeri nuclear test site including the coverage of the used satellite data; **(b)** colored shadow relief map of the study area, **(c)** RGB image acquired on 27. Aug. 2017 (pre-explosion); **(d)** RGB image acquired on 23. Sep. 2017 (post-explosion); the star indicates the location of the sixth nuclear test from the 38 north; the gray triangle indicates the entrance of the test site.; The source data of shadow relief map is shuttle radar topography mission **(a,b)**; the optical RGB images acquired from Pleiades; the figure was generated by the GMT software 5.4.5 version (https://gmt.soest.hawaii.edu/) and GIMP software 2.8.22 version (https://download.gimp.org/mirror/pub/gimp/v2.8/windows/).

Figure 1a showed the study area, which is the North Korea nuclear test site. The topographic height is very complex, since there are the complex mountains around Mt. Mantap (Fig. 1b). The epicenter is close to the peak of Mt. Mantap (2,209 m), which implies that the surface deformation would include both the landslide and explosion components^43,44^. The underground tunnel network from the north portal is connected to five other test sites^2,45^. It means the long and complex underground cavity had been constructed. If the ground stability of the tunnel is poor, it is possible to recognize the deformation pattern of a linear form^1,6,31,46^. Figure 1c,d respectively showed Pleiades high-resolution optical satellite images acquired in pre- and post-explosion. Even though the temporal difference between two optical images was only 28 days, a lot of land cover changes could be found^2^. Especially the vegetation on the top and ridge of Mt. Mantap disappeared, as one can see in Fig. 1c,d.

Table 1 summarized the principal parameters of interferometric pairs used. The ALOS PALSAR-2 stripmap acquisitions from the ascending and descending orbits were used for this study. Each acquisition fully covered the study area (Fig. 1a). The ascending pair was acquired on 29th August, 2017 and 12th September, 2017 and the descending pair was acquired on 31st August, 2017 and 28th September, 2017. The temporal baselines were 14 and 28 days in the ascending and descending pairs, respectively. Since the difference between the two temporal baselines are 14 days, it could be expected that the decorrelation due to the temporal baseline is very low. And also, the perpendicular baselines of the ascending and descending pairs were about − 11 and − 64 m, respectively. The perpendicular baseline of the descending pair was almost six times larger than the ascending pair. Thus, we can expect that the descending measurement will be more affected by topographic effect than the ascending measurement^36,41,47^. The incidence angles of the ascending and descending pairs were about 42.9 and 36.2 degrees, respectively. We can expect that the descending pair had a more severe geometric distortion (i.e. foreshortening effect) due to the relation between the incidence angle and topography ^46,47^.

**Table 1:** Parameters of co-seismic interferometric pairs used in this study.

Parameters	Ascending	Descending
Mode	SM1	SM1
Acquisition date (dd/mm/yyyy)	29/08/2017 12/09/2017	31/08/2017 28/09/2017
Range pixel spacing (m)	1.43	1.43
Azimuth pixel spacing (m)	1.84	2.12
Incidence angle (˚)	42.9	36.2
Heading (˚)	− 9.4	169.9
Perpendicular baseline (m)	− 11	− 64
Temporal baseline (days)	14	28

### Offset tracking measurements

The two co-seismic interferometric pairs were processed by (1) the azimuth common band filtering and sub-pixel-level registration, (2) multiple offset estimation, and (3) initial azimuth and range OT generation using the 3D median filtering. The offset was calculated every 4 pixels in the azimuth and range directions to reduce the computation time. The step of 4 pixels in the offset estimation corresponds to the 4X4 multi-looking in the InSAR method. Thus, the pixel spacing in the measured OT maps was about 7.4 × 8.4 m in azimuth and range directions, respectively. The kernel sizes were designed from 96 × 96 to 256 × 256 pixels with the interval of 32 pixels in both the azimuth and range directions, respectively. Thus, a total of 36 OT measurements were calculated by using the kernel sizes of 96 × 96, 96 × 128, 128 × 96, 96 × 160, 160 × 96, 128 × 160, 160 × 128, …, 224 × 256, 256 × 224.

**Figure 2:**
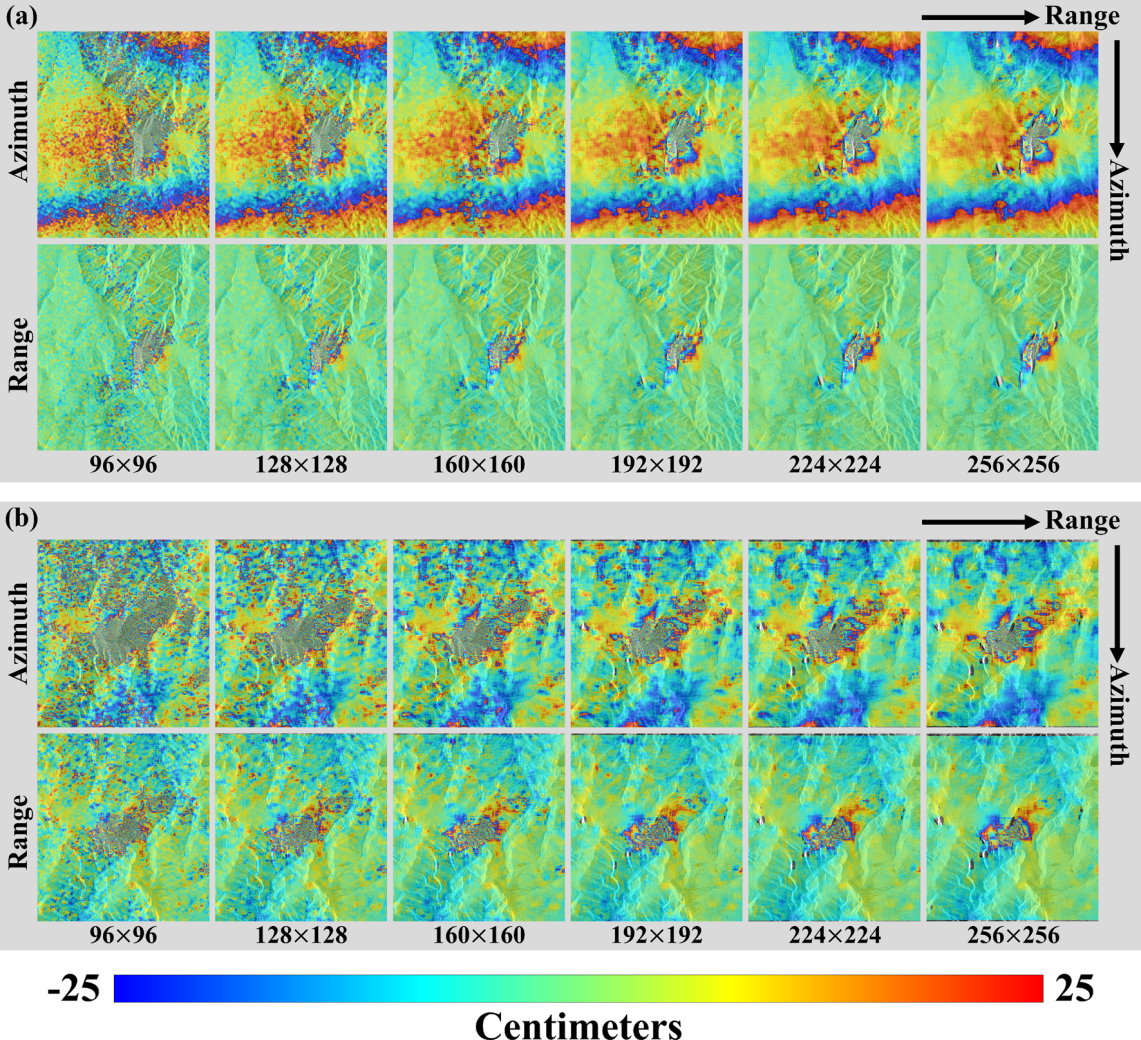
Azimuth and range offset tracking measurements according to multiple kernel size. **(a)** from an ascending pair and **(b)** a descending pair; the magnitude of each measurement was mapped by wrapping 50 cm/cycle; The figure was generated by the GMT software 5.4.5 version (https://gmt.soest.hawaii.edu/) and GIMP software 2.8.22 version (https://download.gimp.org/mirror/pub/gimp/v2.8/windows/).

Figure 2 illustrates the multiple OT maps estimated from the square kernels. Figure 2a,b respectively show the multi-kernel OT measurements from the ascending and descending pairs (20170829_20170912 and 20170831_20170928). As you can see Fig. 2, as the kernel size is smaller, the measurement accuracy is much lower but the spatial resolution is much higher while the measurement accuracy is higher, but the spatial resolution is lower, as the kernel size is larger. It means that the measurement accuracy and spatial resolution have a trade-off relation^24,36–38^.

You can see the long-wavelength patterns in the azimuth OT maps from ascending and descending pairs (20170829_20170912 and 20170831_20170928) as seen in Fig. 2. It was caused by ionospheric effects^47^. The patterns must be reduced by an ionospheric correction method. And, the ascending range OT maps were almost flat except the deformation area (Fig. 2a) while the descending range maps were variational according to topographic height (Fig. 2b). Since the perpendicular baseline of the descending pair was about 6 times longer than that of the ascending pair, the descending pair is about 6 times sensitive to topographic variation. Thus, Fig. 2b shows that the topographic effect must be corrected in the descending range maps using an existing digital elevation model (DEM). We first generated initial offset maps by applying the 3D median filter to the 36 offset measurements. The 3D median filter can efficiently suppress the noise effect from the multiple offset measurements. The 3D median filter was designed to calculate the median value from the kernel sizes of 3X3X36 in the azimuth, range and measurement directions.

**Figure 3:**
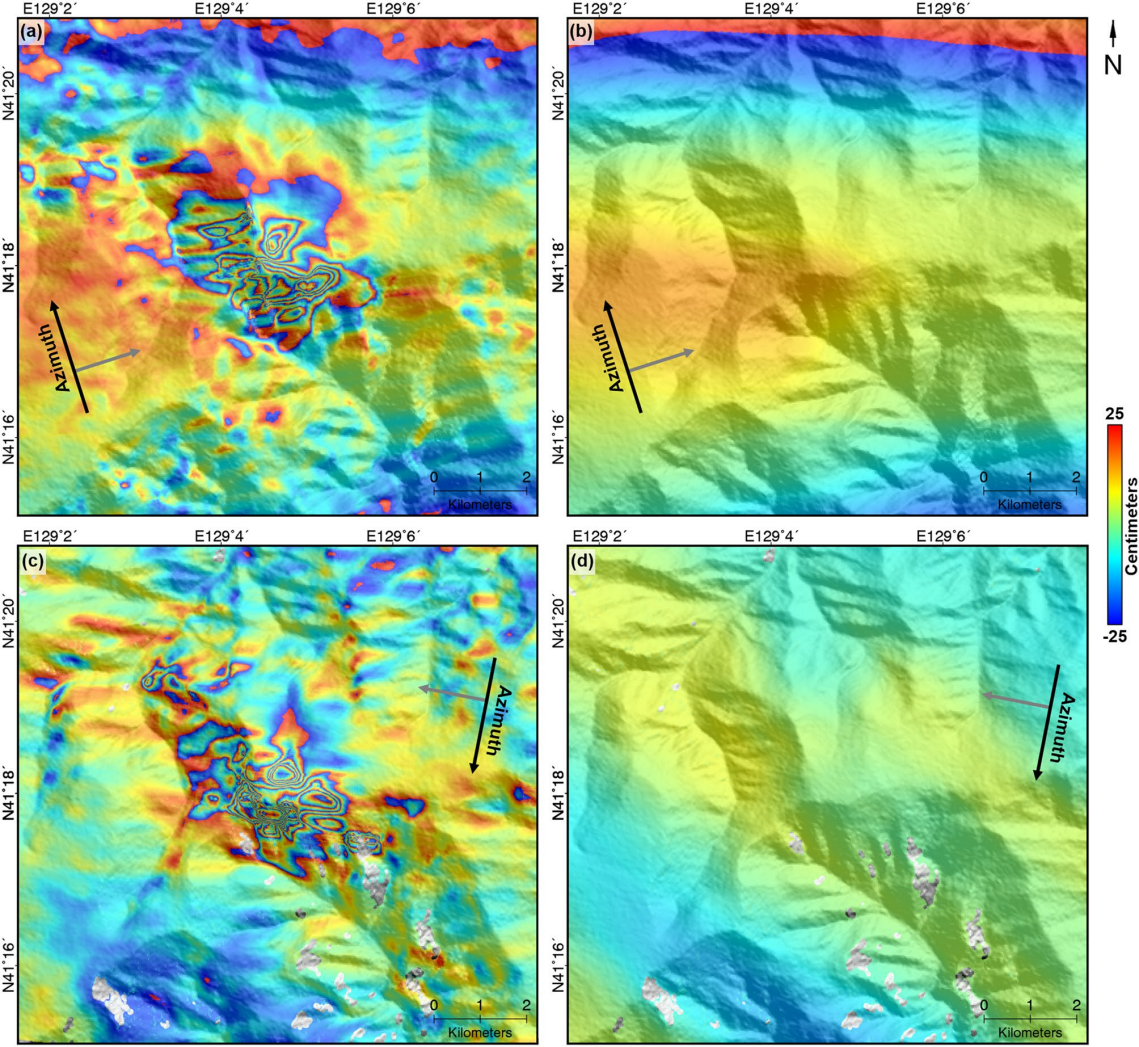
Initial azimuth offset measurements of **(a)** the ascending and **(c)** descending pairs and **(b)** the ascending and **(d)** descending ionospheric maps extracted from **(a)** and **(c)** using the directional median filter. The offset measurements were represented by the fringe of 50 cm/cycle; The figure was generated by the GMT software 5.4.5 version (https://gmt.soest.hawaii.edu/) and GIMP software 2.8.22 version (https://download.gimp.org/mirror/pub/gimp/v2.8/windows/).

As aforementioned, the error components of SAR-derived deformation maps such as topographic and ionospheric effects should be corrected or reduced^11,25,47^. Figure 3 shows initial azimuth OT maps and its ionosphere component maps, which were calculated from the directional median filtering^24,25,38,47,48^. Ionospheric effect shows azimuth streaking on the SAR-derived deformation maps in general. Applying large-kernel directional median filter along streaking direction can mitigate high-frequency signal (such as localized deformation, OT measurement errors) and generate ionospheric phase screen^24,25,38,47,48^. To estimate the ionospheric component, the steep deformation area on Mt. Mantap was first masked out, and then the directional median filters were designed for the ascending and descending azimuth OT maps, respectively (Fig. 3b,d). The main parameters for designing directional median filter are kernel size and rotation angle. The kernel size indicates the height and width of a moving window in pixel numbers, calculating ionoshperic components of each pixel. The rotation angle is determined by the direction of the ionospheric streak pattern. The detailed description and diagram of these two parameters can be found on a previous study^24^. The kernel size of 813 × 153 were selected for the both ascending and descending azimuth OT maps. In addition, the rotation angles of 70.2 deg and 51.6 deg were experimentally selected for the ascending and descending azimuth OT maps respectively. The fact that the standard deviations of ionospheric component maps were about 11.4 and 5.2 cm for ascending and descending pairs tells that the ionospheric effects were more severe in the ascending pair. The standard deviations, which were calculated in the non-deformation area, of the ascending and descending azimuth OT maps before and after mitigating the ionospheric distortion were changed from about 18.0 and 9.0 cm to about 4.2 and 7.0 cm, respectively. It means that the ionospheric distortion was efficiently reduced.

After removing these ionospheric errors, topographic correction was applied to the initial OT maps. The correction was performed by adopting a linear regression model between elevation and measured offsets. More detailed descriptions of topographic effect and correction in OT maps can be found in Baek et al. (2018)^36^.

### Three-dimensional deformation retrieval

**Figure 4:**
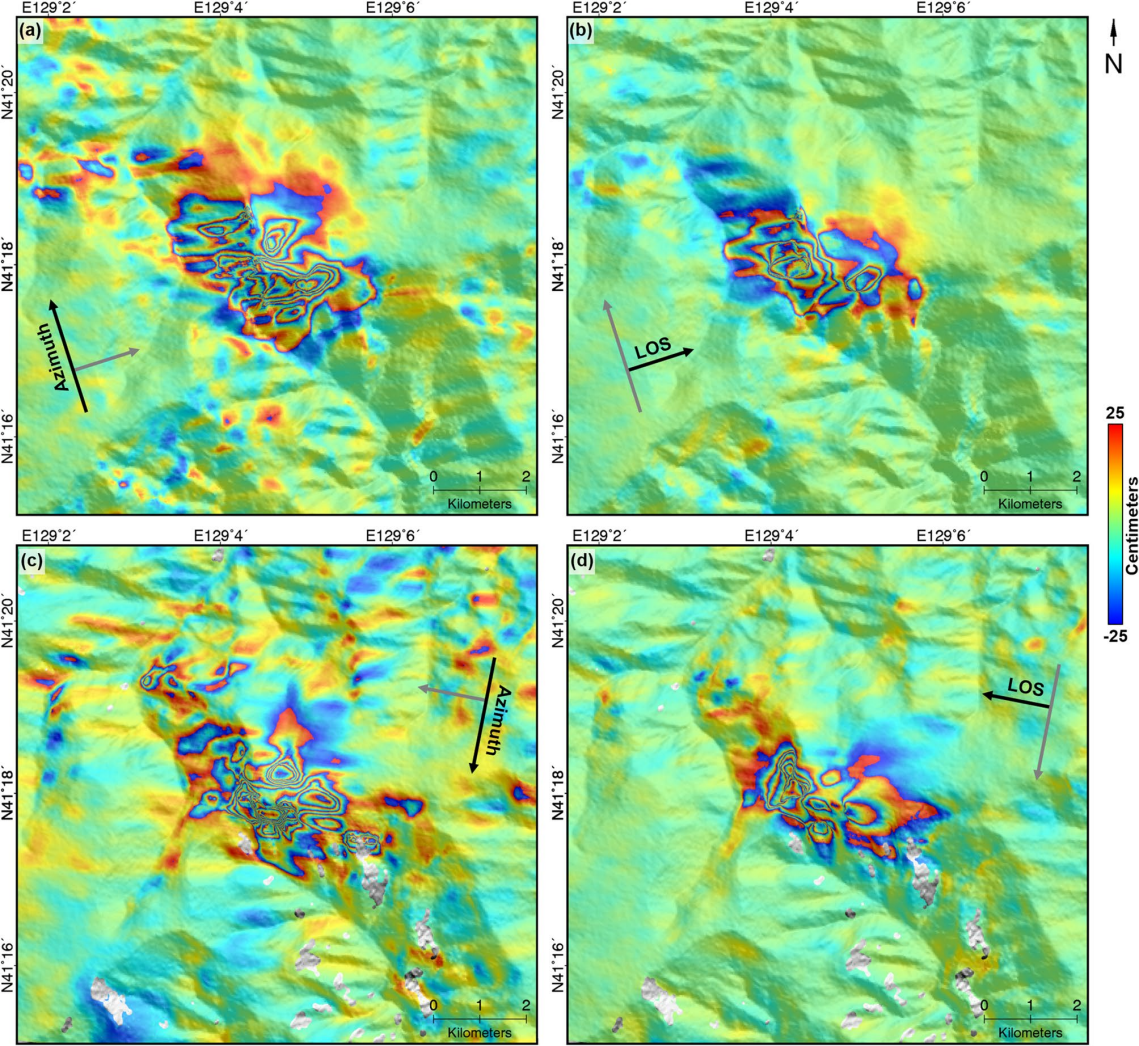
Final offset tracking (OT) maps: **(a)** ascending azimuth OT map, **(b)** ascending range OT map, **(c)** descending azimuth OT map, and **(d)** descending range OT map. The offset measurements were represented by the fringe of 50 cm/cycle; The figure was generated by the GMT software 5.4.5 version (https://gmt.soest.hawaii.edu/) and GIMP software 2.8.22 version (https://download.gimp.org/mirror/pub/gimp/v2.8/windows/).

Figure 4 shows the final OT maps generated by mitigating error components from initial OT maps. We can clearly see a similiar deformation pattern in the azimuth and range OT maps (Fig. 4). In the azimuth OT maps, the Northern and Southern parts of the maps respectively show the northward and southward deformations clearly. Similially in both the range OT maps, the deformation of expansion in east–west direction were definetely recognized. The azimuth OT maps were much noisier than the range OT maps because the measurement performance of the range OT is much better than that of the azimuth OT^47,49^. The maximal measured deformation was about − 266.9, − 318.3, 482.6, and 328.9 cm in the ascending azimuth and range OT maps and the descending azimuth and range OT maps, respectively (Fig. 4a–d). The maximal magnitudes were different because (1) the acquisition geometry (heading and incidence angle) was different and (2) the amount of compaction and landslides could be different due to the different temporal baseline of each pair^1^. The deformation pattern with the radial shape were predictable from the four measurements. However, for the better understanding the deformation pattern, it is important to retrieve a 3D deformation field from the four measurements^41^.

**Figure 5:**
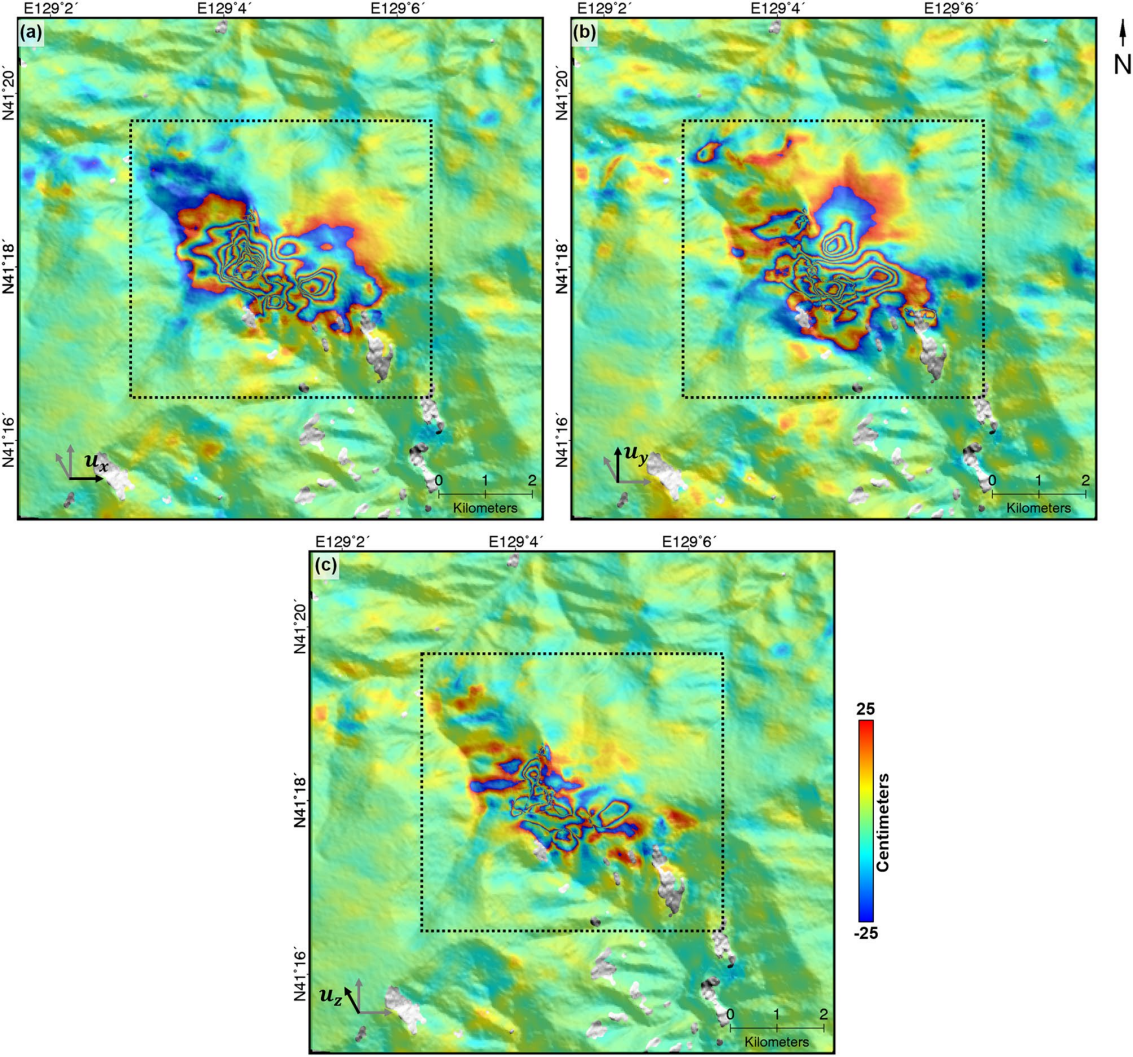
Three-dimensional surface deformation field retrieved by integrating the four final offset measurements of Fig. 4; **(a)** east, **(b)** north, and **(c)** up components of surface deformation. Positive signals represent eastward, northward, and upward movements; The measurements were mapped by wrapping 50 cm/cycle. The inside and outside of box are respectively supposed as deformed and non-deformed area for the validation; The figure was generated by the GMT software 5.4.5 version (https://gmt.soest.hawaii.edu/) and GIMP software 2.8.22 version (https://download.gimp.org/mirror/pub/gimp/v2.8/windows/).

Figure 5 shows the 3D surface deformation field retrieved by combining two range and two azimuth OT measurements shown in Fig. 4. The eastward, northward and upward directions are respectively positive in the deformation maps of Fig. 5a–c. The radial deformation pattern are clear in both the East and North directions (see Fig. 5a,b). However, the deformation pattern in the up direction was not radial but complex and irregular (see Fig. 5c), because the sixth nuclear test-related deformation included the components of uplift, landslides, collapse and compactions^1^. The maximal deformation is about − 491.0, − 343.0 and 165.6 cm in the east, north and up directions, respectively. The maximal horizontal deformation reached about 514.9 cm. The magnitude of horizontal deformation was larger than the known results^1^ (3.5 m) because the estimated 3D maps have a higher resolution and precision via the MKOT approach^1,24,25^.

The in-situ measurements was almost never released, and hence the validation with a in-situ confident data on this area is realistically impossible^8^. However, the validation is an important process to enhance the confidence of the measured 3D deformation field. In case of no confident validation data, the measurement performance has been estimated by calculating the mean and standard deviation of stable^24,25^. Thus, to validate the measurment performance of the measured 3D deformation, the mean and standard deviation of each component of the 3D deformation were calculated in the non-deformed area, which was selected as the outside of dotted black box on Fig. 5 in the consideration of the deformation size and terrain effects such as the slope and aspect.

**Figure 6:**
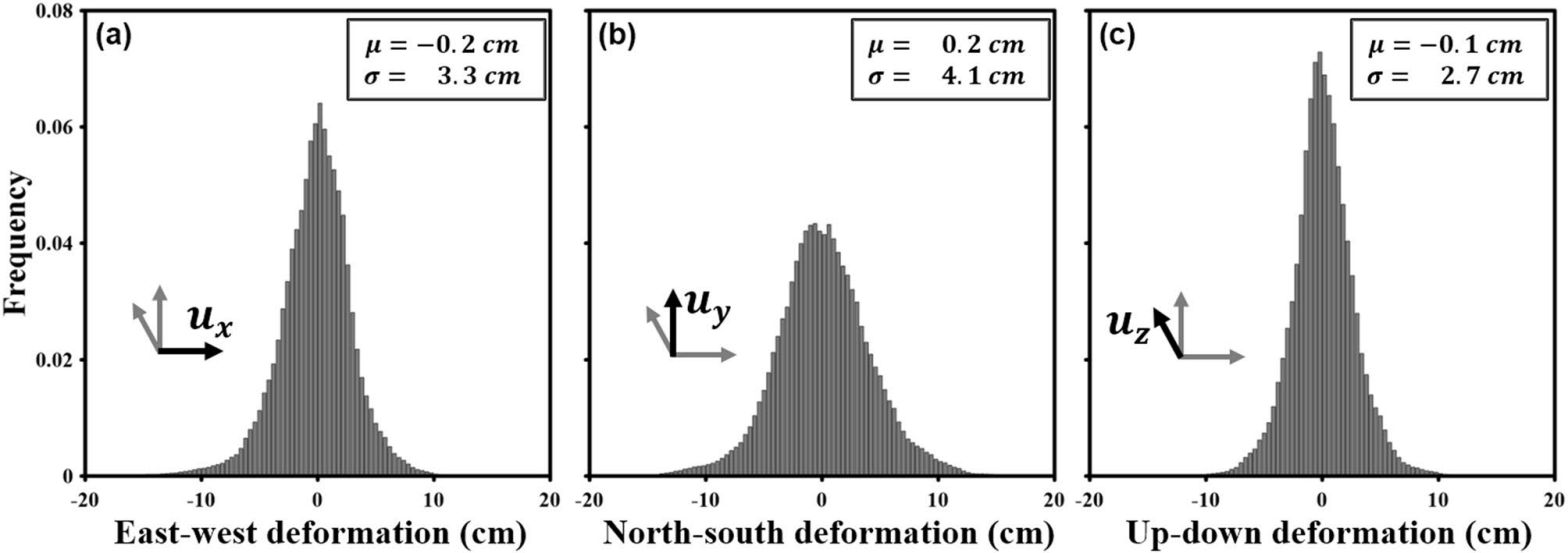
Histograms of deformations in the **(a)** east, **(b)** north and **(c)** up directions. The deformations outside of the dotted black box on Fig. 5 were used for this calculation; The figure was generated by the GMT software 5.4.5 version (https://gmt.soest.hawaii.edu/) and GIMP software 2.8.22 version (https://download.gimp.org/mirror/pub/gimp/v2.8/windows/).

Figure 6 represents the histograms of the measured deformations in the east, north and up directions on non-deformed area. The means in the east, north and up directions were about − 0.2, 0.2 and − 0.1 cm, respectively. This indicates that the deformations in all the directions were not biased^49^. The standard deviations were about 3.3, 4.1 and 2.7 cm in the east, north and up directions, respectively. The standard deviation in the north direction was slightly larger than others because the north component is largely dependent on the azimuth measurements (1) that were contaminated by ionospheric effects (despite to the ionospheric correction, the precision cannot be better than that of non-ionosphere-contaminated measurements^38,41^) and (2) that had a lower precision than the range measurements^47,49^. This histogram analysis indicates that the measurement performance of the retrieved 3D deformations is reliable with centimeter precision.

### Horizontal deformation

**Figure 7:**
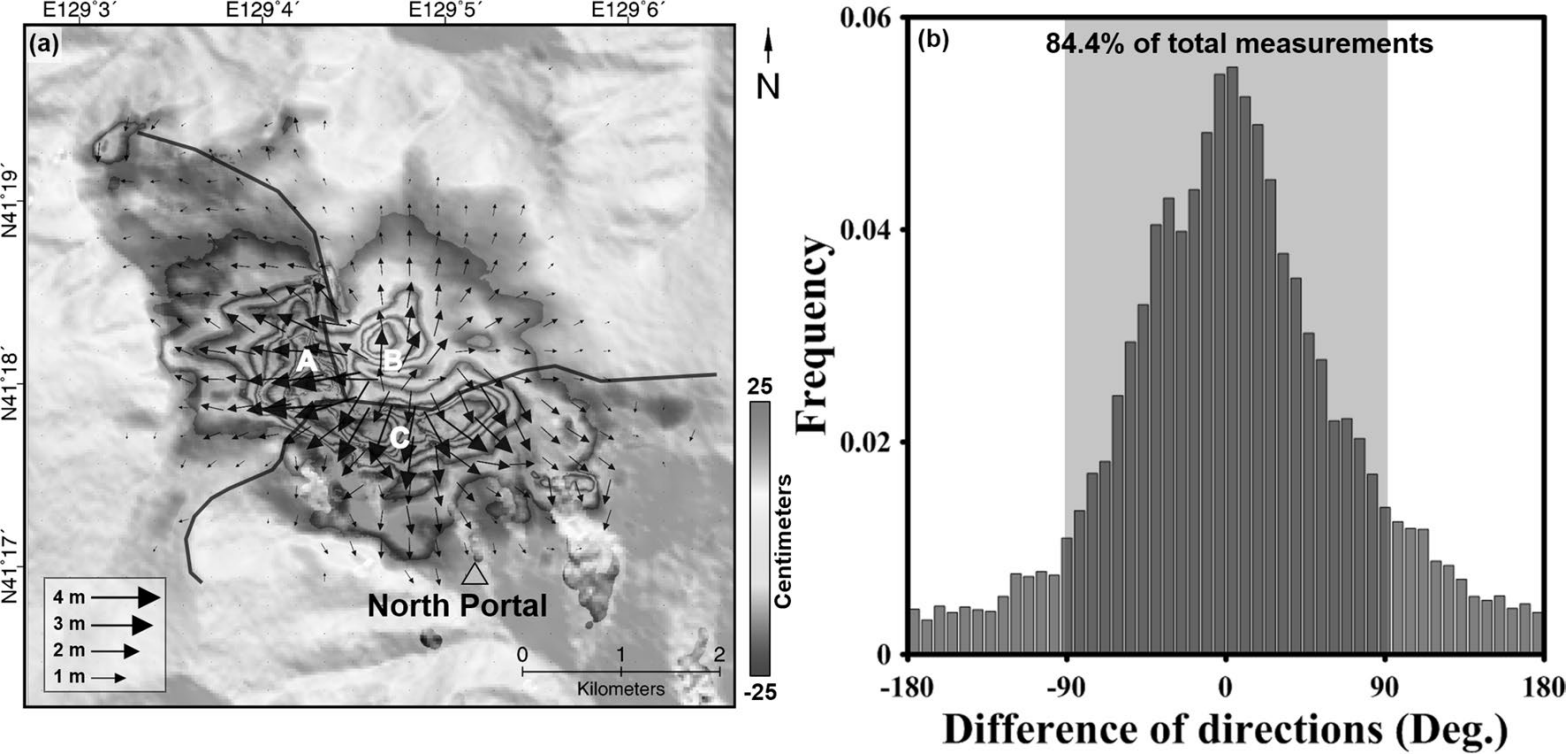
**(a)** Horizontal surface deformation fields and **(b)** histogram calculated from the angular difference between the horizontal deformation vector and terrain aspect vector. In the horizontal deformation field, the color represents the magnitude of the horizontal deformation and the black arrows indicates the direction and magnitude of the horizontal deformation; The figure was generated by the GMT software 5.4.5 version (https://gmt.soest.hawaii.edu/) and GIMP software 2.8.22 version (https://download.gimp.org/mirror/pub/gimp/v2.8/windows/).

Figure 7a shows the horizontal deformation of the 3D measurements. The black arrows indicate the direction and magnitude of the horizontal deformation. The solid black line shows the ridge of Mt. Mantap. The deformation directions could be roughly classified as westward, northward and southward based on local maxima, which were marked as A, B, and C in Fig. 7a, respectively. Moreover, all the A, B and C in Fig. 7a were located in the middle of the ridges. The magnitude and direction of the horizontal deformations with respect to the mountain ridges indicate that the horizontal deformation would be largely related to topography of Mt. Mantap.

Thus, the direction of the measured horizontal deformation was compared with the topographic aspect calculated from 1 arc-second shuttle radar topography mission (SRTM) DEM. Figure 7b shows the histogram of angular difference between horizontal deformation vector and terrain aspect vector. Consequently, the mode was formed around 0 deg. Moreover, approximately 84.4% of the measurements were distributed in the range of − 90 to 90 deg. This result indicates that the horizontal deformation was profoundly affected by the slope and aspect of the topography. It means the horizontal deformation can be decomposed to the explosion-related radial deformation and topography-related landslide.

### Vertical deformation

**Figure 8:**
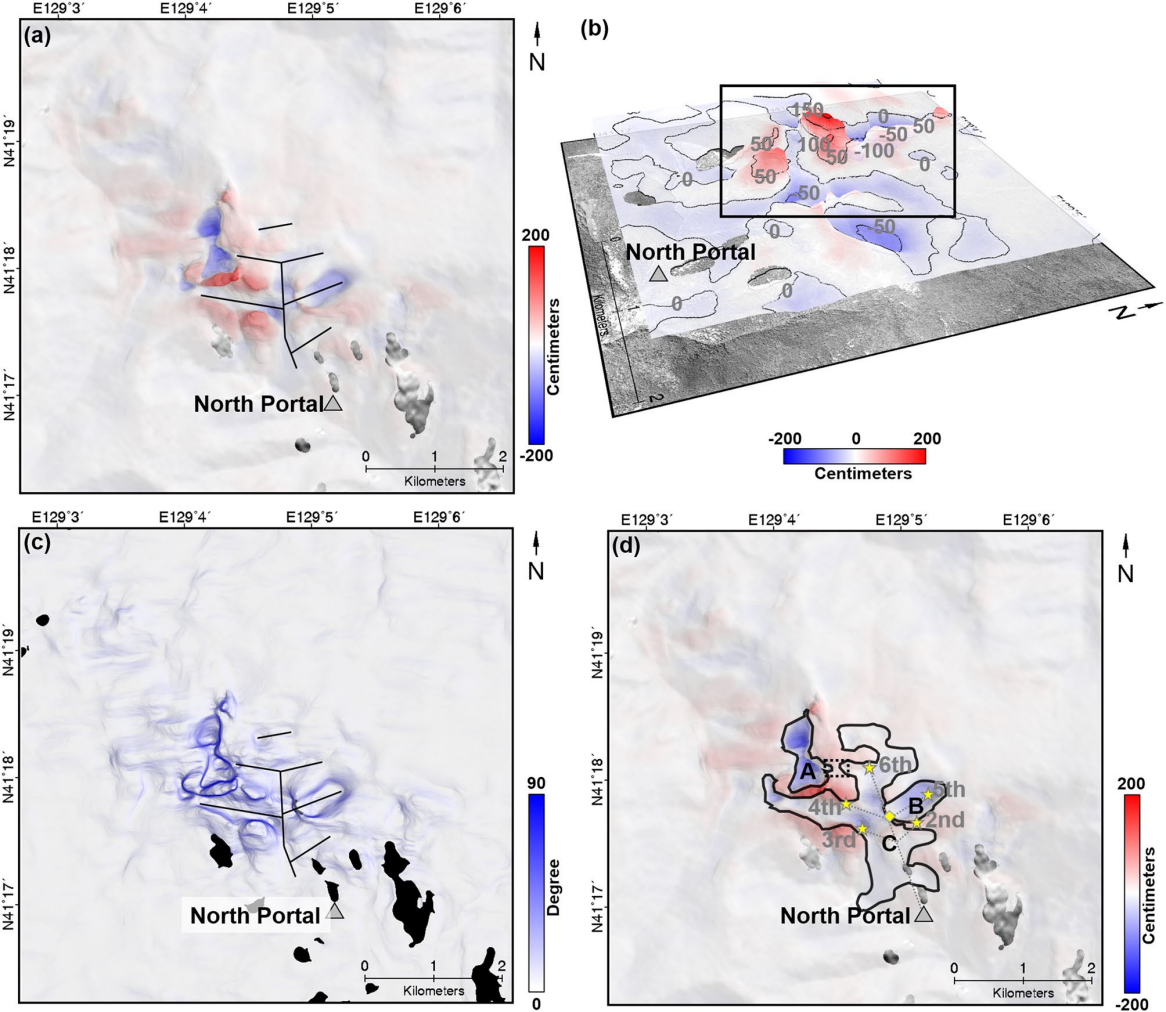
Vertical surface deformation. **(a)** Measured vertical surface deformation; **(b)** Three-dimensional plotting of vertical surface deformation. The height and color indicate the up deformation. Numerical digits belong to contour lines in centimeter scale. **(c)** Calculated slope from the magnitude of vertical deformation. **(d)** measured vertical surface deformation with possible collapse area, A, B and C (black polygons); Black solid line of **(a)** and **(c)** indicate linear subsidence patterns of study area; The stars and dotted gray lines on **(d)** are presented past nuclear test sites and underground tunnels from 38 north^54^; And the yellow diamond on **(d)** indicates the location of second events suggested by Wang et al. (2018)^1^; The figure was generated by the GMT software 5.4.5 version (https://gmt.soest.hawaii.edu/) and GIMP software 2.8.22 version (https://download.gimp.org/mirror/pub/gimp/v2.8/windows/).

Figure 8a shows the vertical deformation map in a scale of range between − 200 and 200 cm. It was well known that the deformation caused by underground nuclear explosion showed the pattern of isotropic expansion. However, the measured vertical deformation could not explain the isotropic deformation pattern. Relatively a severe vertical deformation was observed in the left part from the center of study area. In addition, most importantly, a spatially linear subsidence pattern was identified on Mt. Mantap. The black solid lines indicate the linear subsidence pattern (see Fig. 8a). In many cases, unstable artificial tunnels or a void space cause a spatially linear subsidence^6,31,46,50^. The underground tunnel network had been constructed from the north portal to the nuclear test positions under Mt. Mantap^45,51^. Thus, we can assume that the relatively severe vertical deformation and spatially linear subsidence pattern were caused by underground artifical void space such as tunnel.

Figure 8b showed three-dimensional plotting of the vertical deformation. The uplift and subsidence coexisted complexely. In the black box, the uplift of more than 150 cm and the subsidence of more than 100 cm were measured coincidencely. Since the study area underwent the expansion by explosion, landslides, collapse and compaction successively^1^, the components were mixed in the measured deformation. Thus, if the magnitudes of collapses or compactions are locally smaller than that of expansion, the vertical deformation would show a positive value (uplifts) irrespective of downward movements. On the other hands, if the downward components are larger than the upward component, the vertical deformation would have a negative value (subsidence), but it can be underestimated due to the expansion component.

The collapse area was decided from the vertical deformation by calculating the deformation rate. Figure 8c shows the vertical deformation change rate map. The deformation rate map was calculated in the same way as calculating the slope from DEM. Thus, the rate map ranged from 0 to 90 degree and complete blue means 90 deg while complete white is 0 deg. In the areas of most extreme change, the rate reached almost 84 degree. The spatially linear pattern could be easily recognized in Fig. 8c. Especially, a large change was recognized in the left of the image center. We decided the subsidence area using the magnitude, direction and rate of the vertical deformation.

The subsidence areas were marked in Fig. 8d. Figure 8d shows the tunnel-related subsidence area aforementioned (solid black lines), the implosion location suggestd from Wang et al. 2018 (yellow diamonds)^1^, and the location of tunnels (grey dotted lines) from 38 North^1,2,45^. The subsidence areas were divided into the A, B and C zones. In the zone C, there were a spatially linear pattern extending to west, north and south from the location of implosion^1^. Although the uplift signal were measured between the zones B and C, it is possible to have a connection between the zones B and C. Likewise, it has a high possibility that the zones A and C would be connected although the subsidence pattern was partially discontinued in the area of dotted black box on Fig. 8d. These continuities would seem to be significantly associated with the underground tunnel networks.

The zones A, B and C have areas of about 0.3765, 0.3146, and 2.0477 km^2^, respectively. The areas of the zones A and B were similar, but the area of the zone C was about 6 times larger than both the zones A and B. And we also calculated the volume change on the zones A, B and C to approximately estimate the total amount of the collpase. Before the volume change calculation, the mean deformation of the each boundary was calculated to set a reference point of the deformation because the vertical deformation included complex up and down deformation components^1,49^, and the volume change was calculated by compensating the reference vertical deformation. The volume changes in the zones A, B and C were about − 2.58E + 05, − 1.04E + 05 and − 4.28E + 05 m^3^, respectively. The volume changes in the zones A, B and C are same amount of homogeniously subsiding 36.2, 14.5 and 60.0 m in the area of a soccer field, respectively. Despite the area difference of A, B and C, the volume change of the zone C was about 1.7 and 4.1 times greater than the zones A and B. Moreover, the mean subsidences from the reference vertical deformations were about − 68.6, − 32.9 and − 20.9 cm in the zone A, B and C, respectively. From the results, it can be easily recognized that most severe subsidence occurred in the zone A. It should be noted that the zone A must be considered as a severe collapse, or if not, at least be monitored to check long-term geological stability^46^.

The location of the tunnel and tunnel-related subsidence area show a discrepancy. This is because we manually situated the locations of the tunnel and test sites using the photo from 38 North because we didn’t have documented information about the cordinates of the underground structures^45,51^. In other words, the branching patterns of the tunnels were similar, but the locations were not exactly the same^45,51^. The discrepancy is still controversial, and hence additional analysis needs to be performend by using various measurements or documentations. Despite these discrepancies, we believe that our surface displacement measurement could suggest a possible collapse of the tunnel under Mt. Mantap .

## Discussion

Radar data have been successfully used to monitor surface deformation because they have made it possible to measure the surface deformation changes with submeter accuracy. The phase information of the radar data enables the precise measurement. However, the phase information has an inevitable disadvantage that it cannot be applied to large and complex surface deformation areas since the measurement values of adjacent pixels must be present at half the radar wavelength. Normally, the radar wavelengths are about 3, 6 and 24 cm in X-, C- and L-band radar data respectively. If deformation difference among adjacent pixels exceeds half of the radar wavelength, no valid radar phase information can be obtained. In this case, instead, the intensity information of the radar data has been widely used. Conventional methods have low precision over 10 cm and low spatial resolution over 100 m, but they are nevertheless employed to estimate the surface deformation changes from the intensity as those methods can measure the large and complex surface deformation.

The surface deformation related to North Korea’s sixth nuclear test was very large and complex, and it consisted in explosions, collapse, compaction and landslides^1^. Thus, the decomposition of the deformation is the most crucial step in esimating the explosive yield from the nuclear detonation. If the step of decomposition is ignored, the explosive yield is most likely to be either overestimated or underestimated. In spite of that, the estimated explosive yield of 191 kilotonnes of TNT equivalent and the source depth of 450 m has been already reported without the necessary deformation decomposition^1^. It is fairly reasonable to expect the estimated explosive yield to be either overvalued or undervalued in that the surface deformation measured in the previous studies had low accuracy and spatial resolution. Unavoidably, the measured deformation only showed an overall deformation pattern in the absence of a decomposition process.

As discussed above, one of the most essential things in monitoring nuclear tests is to recognize the detailed local deformation pattern. The detailed local deformation pattern permits not only analyzing the spatial distribution of the deformation components but also decomposing the deformation components. In this study, the precise local deformation was measured by the MKOT method. The MKOT measurements have been well known to deliever distinctly improved performance such as several centimeters of accuracy and tens of meters of spatial resolution. When 3D deformation field was retrieved from the MKOT measurements, we could find (1) that the horzontal deformation was mainly mixed by the deformation components from explosions and landslides and (2) that the vertical deformation mostly included the deformation components of explosions and collapses.

The horizontal deformation vector showed the explosion-induced isotropic deformation pattern. The horizontal deformation became larger closer to the epicenter of the sixth nuclear test, and the deformation concentrically decreased as the deformation areas moved far away from the epicenter. However, since the epicenter is adjacent to the top of Mt. Mantap, the topographic slope vector related to landslides showed a horizontal deformation pattern similar to an explosion-induced deformation pattern. Unfortunately, separation of horizontal components between the landslide-induced and explosion-induced deformation vectors is not easy because of similarities in their deformation patterns. The nuclear test facility might have been designed the way it was, taking these deformation similarities into consideration. Anyway, it is true that the deformation similarity may lead to overestimation or underestimation of the nuclear explosive yield.

The vertial deformation did not show the isotropic deformation induced by the explosion because the collapse areas were very large^1^. The maximum collapse came to over 100 cm, and it would even amount to 250 cm if the extended uplift of 150 cm was counted. The large collapse can be caused by either natural underground caves or artificial underground facilities. However, the collapse under consideration were way too big to say that they resulted from natural underground caves. This collapse also has a linear subsidence pattern as shown in Fig. 8. Therefore, it was most likely to be brought about by artificial underground facilities. Moreover, the estimated epicenter of the second, third, fourth, fifth and sixth nuclear tests, and the approximate locations of underground tunnels were all around the collapse areas (B and C of Fig. 8d). This may give us some useful clue to decide whether or not the Punggye-ri nuclear test site can be reusable.

The second earthquake after a main shock would be very likely caused by this collapse. However, no such underground facilites in the area A were identified from the large scale topographic map that the North Koreans displayed^2,45^. In our results, the largest collapse was found in the collapse area A in Fig. 8d. The subsidence in the collapse was about 70 cm on average and about 120 cm maximum. Those amount of surface deformation should not be overlooked. We carefully conjecture about the collapses were caused by the failures of underground facility and it caused the second earthquake. Thus, it would be seem that it has had lost the function for good, although we don’t know the exact role of the facility under the extent of area A.

A large scale of the collapse and compaction have barred the vertical deformation from being used to estimate the nuclear explosive yield. The explosion caused uplifts of the test sites, after that a collapse and compaction were followed^1^. Those serial process would attenuate the explosion-directly-related deformation components. In other words, the measured vertical surface deformation is underestimated to estimate explosive yield. In spite of that, the explosive yield has been estimated without excluding the vertical deformation^1^. The estimated yield would be most likely underestimated. To estimate the nuclear explosive yield in more accurate, it will be necessary to set aside the vertical deformation for the yield estimation.

## Methods

Wang et al. (2018) reported that the horizontal and vertical deformations induced by the 2017 North Korea nuclear test were maximally about 3.5 and 0.5 m, respectively and the implosion would contribute to only local area^1^. As aforementioned, the vertical deformation mapping with high spatial resolution is essential to distinguish the collapse component caused by explosion from the deformation. The OT method utilizes the intensity cross-correlation between master and slave single look complex (SLC) images. It has been widely applied to retrieval 3D surface deformation in case that the phase signal was decorrelated where a surface deformation is too steep due to large and complex deformation^23,24,36,42,52^. However, the traditional OT method has a trade-off between the spatial resolution and precision of measurements with respect to the offset-estimation kernel size^24,25,36,37^. To overcome the drawback, the MKOT method was applied to the 3D deformation measurement^24,25,36,37^. MKOT is the method to find representative displacements from the statistical properties of the multiple traditional OT measurements, which has a different spatial resolution and precision, by increasing kernel sizes (multi-kernel)^37^. MKOT can generate an optimal deformation map because the measurement noises can be successfully suppressed by averaging multiple measurements and the spatial resolution can be preserved in the procedure of determining representative value^24,25,36,37^. In addition, an error mitigation process is applied to the OT map to reduce the errors before mapping 3D surface deformation, because the OT map is affected by the topographic and ionospheric errors ^24,36,41^.

**Figure 9:**
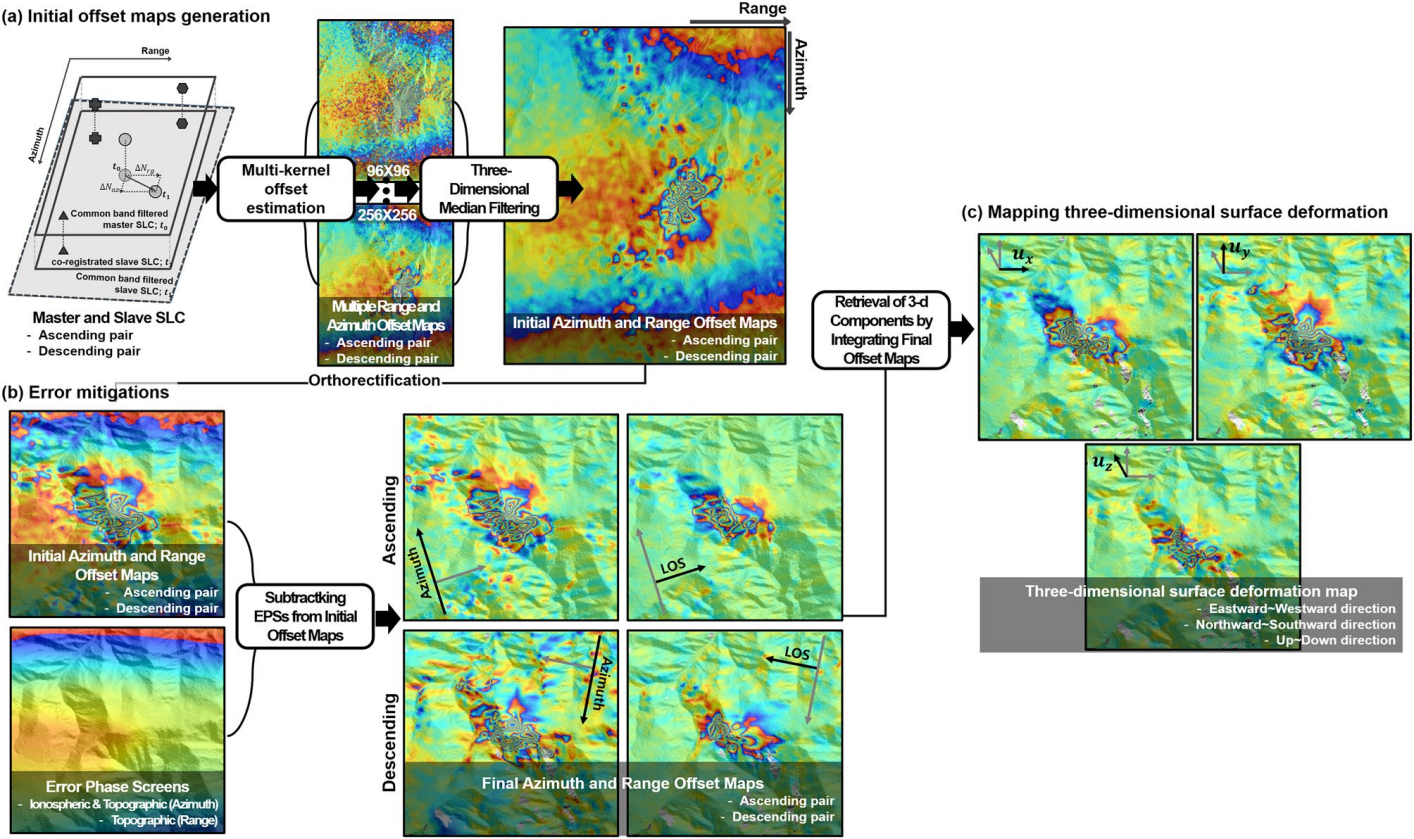
Workflow of this study; overall data flow is categorized into three parts: **(a)** initial offset maps generation; **(b)** error mitigation; **(c)** three-dimensional surface deformation retrieval; The figure was generated by the GMT software 5.4.5 version (https://gmt.soest.hawaii.edu/) and GIMP software 2.8.22 version (https://download.gimp.org/mirror/pub/gimp/v2.8/windows/).

Figure 9 describes a systematic data process used for this study. The main processing steps are composed of the following three parts:

- Generating initial OT maps^24,41^.
- Conducting error mitigation^24,36–38,41^.
- Mapping 3D surface deformation^16,40,42^.

The three processing steps are applied to ascending and descending pairs. The initial OT maps are generated by the MKOT process, which consists of three main steps: (1) co-registration of master SLC image and slave azimuth common band-pass filtered SLC image ^11,24,25,37,38^, (2) estimation of multiple offset measurements using multi-kernels, and 3) generation of initial azimuth and range OT maps through a 3D median filtering^16,40,42^.

Since the topographic and ionospheric effects can largely degrade the OT measurement precision^36,38,41^, we need to reduce the effects in the error mitigation step. The topographic effect is caused by Earth surface’s topography. It depends on the perpendicular baseline and topographic height^36,41^. Although the perpendicular baseline of the two pairs were relatively short (about − 11 and − 64 m in the ascending and descending pairs, respectively), the topographic effect should be corrected by using an existing DEM because the topographic variation over the study area was complex. Thus, the topographic effect was mitigated by using SRTM DEM. The ionospheric effect is commonly represented with an azimuth-directional streak pattern on OT maps as well as SAR interferograms^24,38,48,49,53^. Since the ionospheric effect makes the azimuth and range OT maps distorted, it should be mitigated. In this study, the ionospheric distortion was mitigated by a directional median filtering^24,38,48^.

One azimuth and one range OT maps are generated from an interferometric pair via the above process. One ascending pair and one descending pair should be processed to retrieve 3D deformation, and hence at least two azimuth and two range OT maps must be generated from different geometries. In this study, we created two azimuth and two range OT maps from the ascending and descending orbits and retrieved a 3D surface deformation field from the four offset maps^14,41^.

## Data Availability

The Three-Dimensional Surface Deformation Field Related to the 2017 North Korea Nuclear Test can be downloaded online (https://doi.org/10.5281/zenodo.3540854 and https://cafe.naver.com/uosrs/289).

## Code availability

The source code is available from the corresponding author upon reasonable request.

## References

[1] Wang, T. *et al.* The rise, collapse, and compaction of Mt. Mantap from the 3 September 2017 North Korean nuclear test. *Science.* **361**, 166–170 (2018). 10.1126/science.aar7230 29748323

[2] 38 North. *38 North*. *North Korea’s Punggye-ri Nuclear Test Site: Satellite Imagery Shows Post-Test Effects and New Activity in Alternate Tunnel Portal Areas* https://www.38north.org/2017/09/punggye091217 (2018).

[3] United States Geological Survey. *M 6.3 Nuclear Explosion—21 km ENE of Sungjibaegam, North Korea.**United States Geological Survey.* https://earthquake.usgs.gov/earthquakes/eventpage/us2000aert#executive (2017).

[4] Yu H, Yuan Y, Bobet A (2017). Seismic analysis of long tunnels: A review of simplified and unified methods. Underground Space..

[5] Towhata, I. *Geotechnical Earthquake Engineering* (1st ed.) 265–266 (Springer Series in Geomechanics and Geoengineering, 2008).

[6] Valentino, B. *Application of InSAR to Salt Mine Subsidence.* Master Thesis (Cornell University, Ithaca, 2016).

[7] Plattner C, Wdowinski S, Dixon TH, Biggs J (2010). Surface subsidence induced by the Crandall Canyon Mine (Utah) collapse: InSAR observations and elasto-plastic modelling. Geophys. J. Int..

[8] Wei M (2017). Location and source characteristics of the 2016 January 6 North Korean nuclear test constrained by InSAR. Geophys. J. Int..

[9] Zhao L-F, Xie X-B, Wang W-M, Hao J-L, Yao Z-X (2016). Seismological investigation of the 2016 January 6 North Korean underground nuclear test. Geophys. J. Int..

[10] Goldstein RM, Zebker HA, Werner C (1988). Satellite radar interferometry: Two-dimensional phase unwrapping. Radio Sci..

[11] Jung H-S, Won J-S, Kim S-W (2009). An improvement of the performance of multiple-aperture SAR interferometry (MAI). IEEE Trans. Geosci. Remote Sens..

[12] Jung, H. -S., Yun, S. -H., & Jo, M. -J. An improvement of multiple-aperture SAR interferometry performance in the presence of complex and large line-of-sight deformation. *IEEE J. Sel. Top. Appl. Earth obs.* **8**, 1743–1752 (2015).

[13] Jo, M. -J., Jung, H. -S., & Yun, S. -H. Retrieving precise three-dimensional deformation on the 2014 M6. 0 South Napa earthquake by joint inversion of multi-sensor SAR. *Sci. Rep*. **7**, 5485 (2017). 10.1038/s41598-017-06018-0 PMC5511219 28710455

[14] Jo M-J (2015). Measurement of slow-moving along-track displacement from an efficient multiple-aperture SAR interferometry (MAI) stacking. J. Geod..

[15] Jo M-J, Jung H-S, Chae S-H (2018). Advances in three-dimensional deformation mapping from satellite radar observations: Application to the 2003 Bam earthquake. Geomat. Nat. Haz. Risk.

[16] Jo M-J, Osmanoglu B, Jung H-S (2018). Detecting surface changes triggered by recent volcanic activities at Kilauea, Hawai'i, by using the SAR interferometric technique: Preliminary report. Korean J. Remote Sens..

[17] Fujiwara S (2016). Small-displacement linear surface ruptures of the 2016 Kumamoto earthquake sequence detected by ALOS-2 SAR interferometry. Earth Planets Space.

[18] Lee W-J, Lu Z, Jung H-S, Park S-C, Lee D-K (2018). Using a refined SBAS algorithm to determine surface deformation in the long valley Caldera and its surroundings from 2003–2010. Korean J. Remote Sens..

[19] Colesanti C, Wasowski J (2006). Investigating landslides with space-borne synthetic aperture radar (SAR) interferometry. Eng. Geol..

[20] Strozzi T (2005). Survey and monitoring of landslide displacements by means of L-band satellite SAR interferometry. Landslides.

[21] Baek, W. -K., Jung, H. -S., Jo, M. -J., Lee, W. -J., & Zhang, L. Ground subsidence observation of solid waste landfill park using multi-temporal radar interferometry. *Int. J. Urban Sci.* **23**, 406–421, 10.1080/12265934.2018.1468275 (2018).

[22] Calò F (2015). The space-borne SBAS-DInSAR technique as a supporting tool for sustainable urban policies: The case of Istanbul Megacity, Turkey. Remote Sens..

[23] Strozzi T, Luckman A, Murray T, Wegmuller U, Werner C (2002). Glacier motion estimation using SAR offset-tracking procedures. IEEE Trans. Geosci. Remote Sens..

[24] Chae, S. H., Lee, W. J., Jung, H. S., & Zhang, L. Ionospheric correction of L-band SAR offset measurements for precise observation of glacier velocity variations on Novaya Zemlya. *IEEE J. Sel. Top. Appl. Earth Obs.* **10**, 3591–3603 (2017).

[25] Baek W-K, Jung H-S, Chae S-H, Lee W-J (2018). Two-dimensional velocity measurements of Uversbreen glacier in Svalbard using TerraSAR-X offset tracking approach. Korean J. Remote Sens..

[26] Hong S-H (2019). Parallel computing on intensity offset tracking using synthetic aperture radar for retrieval of glacier velocity. Korean J. Remote Sens..

[27] Ferretti, A., Novali, F., Bürgmann, R., Hilley, G., & Prati, C. InSAR permanent scatterer analysis reveals ups and downs in San Francisco Bay area*. Eos Trans. Am. Geophys. Union* **85**, 317–324 (2004).

[28] Ge L, Chang HC, Rizos C (2007). Mine subsidence monitoring using multi-source satellite SAR images. Photogram. Eng. Rem. S..

[29] Zhou X, Chang NB, Li S (2009). Applications of SAR interferometry in earth and environmental science research. Sensors.

[30] Falorni, G., Del Conte, S., Bellotti, F., & Colombo, D. InSAR monitoring of subsidence induced by underground mining operations. in *Proceedings of the Fourth International Symposium on Block and Sublevel Caving*, Vancouver, Canada, October 15–17 (2018).

[31] Rabus, B., Eppler, J., Sharma, J., & Busler, J. Tunnel monitoring with an advanced InSAR technique. in *Radar Sensor Technology XVI,* 83611F, 10.1117/12.918644 (2012).

[32] Vincent P, Larsen S, Galloway D, Laczniak RJ, Walter WR, Foxall W, Zucca JJ (2003). New signatures of underground nuclear tests revealed by satellite radar interferometry. Geophys. Res. Lett..

[33] Carluccio R (2014). A multidisciplinary study of the DPRK nuclear tests. Pure Appl. Geophys..

[34] Wei, M. *Wei Lab*. *North Korea Nuclear Test—Preliminary Results of the September 3, 2017 Nuclear Test*. https://weilab.uri.edu/nk6 (2017).

[35] Wei M, Sandwell DT (2010). Decorrelation of L-band and C-band interferometry over vegetated areas in California. IEEE Trans. Geosci. Remote Sens..

[36] Baek W-K, Jung H-S, Chae S-H (2018). Feasibility of ALOS2 PALSAR2 offset-based phase unwrapping of SAR interferogram in large and complex surface deformations. IEEE Access.

[37] Chae S-H, Lee W-J, Baek W-K, Jung H-S (2019). An improvement of the performance of SAR offset tracking approach to measure optimal surface displacements. IEEE Access.

[38] Baek W-K, Jung H-S (2018). Precise measurements of the along-track surface deformation related to the 2016 Kumamoto Earthquakes via ionospheric correction of multiple-aperture SAR interferograms. Korean J. Remote Sens..

[39] Jónsson S, Zebker H, Segall P, Amelung F (2002). Fault slip distribution of the 1999 M w 7.1 Hector Mine, California, earthquake, estimated from satellite radar and GPS measurements. Bull. Seismol. Soc. Am..

[40] Jung H-S, Lu Z, Won J-S, Poland MP, Miklius A (2011). Mapping three-dimensional surface deformation by combining multiple-aperture interferometry and conventional interferometry: Application to the June 2007 eruption of Kilauea volcano, Hawaii. IEEE Geosci. Remote Sens. Lett..

[41] Jung H-S, Hong S-M (2017). Mapping three-dimensional surface deformation caused by the 2010 Haiti earthquake using advanced satellite radar interferometry. PLoS ONE.

[42] Fialko Y, Simons M, Agnew D (2001). The complete (3-D) surface displacement field in the epicentral area of the 1999 Mw7. Geophys. Res. Lett..

[43] Lee, W. -J., Park, S. -C., Oh, H. -Y., Lee, D. -K., & Jung, H. -S. Surface deformation detection by 6th nuclear test in North Korea using multi-kernel SAR offset method. in *Proceedings of the 20th European Geoscience Union meeting, Vienna, Austria*, April 7–12 (2018).

[44] Lee W-J (2018). Detection of surface changes by the 6th North Korea nuclear test using high-resolution satellite imagery. Korean J. Remote Sens..

[45] Coblentz, D. & Pabian, F. *38 North*. *North Korea’s Punggye-ri Nuclear Test Site: Analysis Reveals Its Potential for Additional Testing with Significantly Higher Yields.* https://www.38north.org/2017/03/punggye031017 (2017).

[46] Milillo P, Giardina G, DeJong MJ, Perissin D, Milillo G (2018). Multi-temporal InSAR structural damage assessment: The London crossrail case study. Remote Sens..

[47] Jung H-S, Hong S-M (2017). Remarks on correcting ionospheric distortions in L-band radar interferometry. Geocarto Int..

[48] Lee W-J, Jung H-S, Chae S-H, Baek W-K (2015). Enhancement of ionospheric correction method based on multiple aperture interferometry. Korean J. Remote Sens..

[49] Jung H-S, Lee W-J (2015). An improvement of ionospheric phase correction by multiple-aperture interferometry. IEEE Trans. Geosci. Remote Sens..

[50] Wang Z, Perissin D, Lin H (2011). Subway tunnels identification through Cosmo-SkyMed PSInSAR analysis in Shangai.

[51] 38 North. *38 North*. *Destruction at North Korea’s Nuclear Test Site: A Review in Photos*. https://www.38north.org/2018/05/punggye052518 (2017).

[52] Rott H, Stuefer M, Siegel A, Skvarca P, Eckstaller A (1998). Mass fluxes and dynamics of Moreno glacier, southern Patagonia icefield. Geophys. Res. Lett..

[53] Meyer F, Bamler R, Jakowski N, Fritz T (2006). The potential of low-frequency SAR systems for mapping ionospheric TEC distributions. IEEE Geosci. Remote Sens. Lett..

[54] Jung H-S, Lee C-W, Park J-W, Kim K-D, Won J-S (2008). Improvement of small baseline subset (SBAS) algorithm for measuring time-series surface deformations from differential SAR interferograms. Korean J. Remote Sens..

